# Food Perception: Taste, Smell and Flavour

**DOI:** 10.3390/foods12193628

**Published:** 2023-09-29

**Authors:** Yanping Chen, Ying Liu, Xi Feng

**Affiliations:** 1 School of Agriculture and Biology, Shanghai Jiao Tong University, Shanghai 200240, China; catherinechenyp@sjtu.edu.cn; 2 College of Food Science and Technology, Huazhong Agricultural University, Wuhan 430070, China; yingliu@mail.hzau.edu.cn; 3 Department of Nutrition, Food Science and Packaging, San Jose State University, San Jose, CA 95192, USA

Flavor is the most important sensory quality in food. Aroma compounds are initially detected by the human olfactory system. During the mastication process, food is further broken down into small pieces, and releases aroma and taste compounds. Recent studies have tried to figure out the mechanisms of flavor formation in different food systems. Additionally, strategies have been developed to control off-putting flavors in food processing and storage. However, the contributions of flavor components to human perceptions and food quality are not fully understood, leaving a significant gap in our knowledge. This Special Issue includes flavor analyses and their interactions with flavor receptors. The interaction of volatile compounds with non-volatile compounds is also discussed. Genetic methods were also used to understand the connection of specific genes to the formation of flavor. In addition, sensory studies were used to better understand consumer behaviors. Lastly, articles applying flavor analysis and sensory tests to product development have also been included.

Off-putting flavors in muscle products are an issue encountered when selling products. Bacteria, the environment, processing method, and storage conditions can all contribute to off-putting flavors in the fish products. Wu et al. systematically reviewed the formation of undesirable flavors in fish, and discussed methods to control off-putting flavors. Deodorization technologies combined with traditional aquaculture management offers an effective solution to minimize off-putting flavors [1].

Recently, different extraction methods have been developed to analyze food flavor profiles. However, each method has its own limitations. For example, Jinhua ham is a traditional Chinese dry-cured ham with characteristic flavor, and Liu et al. investigated different extraction methods to analyze the volatile compounds in Jinhua ham. SPME–GC–TOF/MS was found to be the most effective extraction method to discriminate between samples by age, as it is closest mimic of the human nose [2].

Flavor not only includes aroma, but also taste. Umami is an important taste compound. Dong et al. identified the umami peptides in Hypsizygus marmoreus hydrolysate using LC-MS/MS, and further investigated their binding mechanisms with the T1R1/T1R3 umami receptor using computational simulations [3]. In addition, Gao et al. studied the saltiness-enhancing properties of odorants from Chinese Douchi. Salty foods may be ideal materials from which to select saltiness-enhancing odorants, thereby enabling their utilization in salt reduction [4].

Sensory evaluation is the most direct manner used to study consumer behaviors. Su et al. investigated a descriptive analysis and consumer hedonic test of the sensory qualities of ultra-high-temperature milk, which provided strategies for the identification of the key sensory attributes of the preferred samples [5]. In this Special Issue, An et al. also investigated consumer expectations of flavored water, and potential consumer segments. Their results will be helpful for the promotion of flavored water [6].

Genetic methods have rapidly developed in recent years. Scientists have used genetic editing methods to understand the contribution of specific genes to the flavor of various plants and animals. Tian et al. used genetic analysis strategies (e.g.,: molecular cloning and functional analysis) to investigate the role of DXS and FPS genes from Zanthoxylum bungeanum Maxim. The results indicated that ZbDXS and ZbFPS are positively related to terpene synthesis, a major contributor to volatile organic compounds [7].

Food science is an applied science; food scientists want to use analytical tools to better control food quality and to develop innovative food products. Lotus leaf is considered a medicinal food resource, but only 1% of it is utilized. Ma et al. used lotus leaf as a material to produce tea. The antioxidant activities of lotus leaf tea were also investigated. Meanwhile, highland barley, as a regional food, grows at high altitudes and in cold regions [8]. Wang et al. investigated the flavor profiles of highland barley fermented with different mushroom mycelia. Their results found that fermentation can improve the flavor of highland barley, providing an alternative method of processing highland barley [9]. Lastly, Kim et al. studied the quality of pork patties with the addition of fermented soy sauce during storage. Their results indicated a new method of applying fermented soy sauce to improve the pork patties’ quality and increase their antioxidant properties [10]. Gu et al. investigated the effect of grapes’ harvesting time on wine quality, which provided a solution to determine the best harvest time and winemaking strategies [11].

In summary, this Special Issue, “Food Perception: Taste, Smell and Flavour”, collates the most recent advances in studies of food perception. The editors would like to thank all the authors who submitted their papers to this Special Issue. The editors also want to thank all the anonymous reviewers for their constructive comments. Lastly, the editing team gratefully acknowledge support from MDPI management team.
